# The Nomenclature of Human White Matter Association Pathways: Proposal for a Systematic Taxonomic Anatomical Classification

**DOI:** 10.3389/fnana.2018.00094

**Published:** 2018-11-06

**Authors:** Emmanuel Mandonnet, Silvio Sarubbo, Laurent Petit

**Affiliations:** ^1^Department of Neurosurgery, Lariboisière Hospital, Paris, France; ^2^Division of Neurosurgery, Structural and Functional Connectivity Lab, Azienda Provinciale per i Servizi Sanitari (APSS), Trento, Italy; ^3^Groupe d’Imagerie Neurofonctionnelle, Institut des Maladies Neurodégénératives—UMR 5293, CNRS, CEA University of Bordeaux, Bordeaux, France

**Keywords:** white matter anatomy, association pathways, nomenclature, human brain, dissection, tractography

## Abstract

The heterogeneity and complexity of white matter (WM) pathways of the human brain were discretely described by pioneers such as Willis, Stenon, Malpighi, Vieussens and Vicq d’Azyr up to the beginning of the 19th century. Subsequently, novel approaches to the gross dissection of brain internal structures have led to a new understanding of WM organization, notably due to the works of Reil, Gall and Burdach highlighting the fascicular organization of WM. Meynert then proposed a definitive tripartite organization in association, commissural and projection WM pathways. The enduring anatomical work of Dejerine at the turn of the 20th century describing WM pathways in detail has been the paramount authority on this topic (including its terminology) for over a century, enriched sporadically by studies based on blunt Klingler dissection. Currently, diffusion-weighted magnetic resonance imaging (DWI) is used to reveal the WM fiber tracts of the human brain *in vivo* by measuring the diffusion of water molecules, especially along axons. It is then possible by tractography to reconstitute the WM pathways of the human brain step by step at an unprecedented level of precision in large cohorts. However, tractography algorithms, although powerful, still face the complexity of the organization of WM pathways, and there is a crucial need to benefit from the exact definitions of the trajectories and endings of all WM fascicles. Beyond such definitions, the emergence of DWI-based tractography has mostly revealed strong heterogeneity in naming the different bundles, especially the long-range association pathways. This review addresses the various terminologies known for the WM association bundles, aiming to describe the rules of arrangements followed by these bundles and to propose a new nomenclature based on the structural wiring diagram of the human brain.

## Introduction

“*Unfortunately, nature seems unaware of our intellectual need for convenience and unity, and very often takes delight in complication and diversity*.”

*— Ramón y Cajal (1906)*.

In 1695, Ijsbrand Van Diemerbroeck wrote the following in the second volume of *L’anatomie du corps humain*: “*Descartes in his Traité de l’Homme (1648) tried to establish by several probable conjectures, that the substance of the brain is necessarily all fibrous, and composed of an infinity of filaments which Willis calls small pipes, or flutings. What Descartes saw from the mind’s eyes, Malpighi in his Epistola anatomica de cerebro ad Fracassatum (1665) has demonstrated it by those of the body. Actually, he writes that by means of the microscope he has very often observed in the brains of oxen, and other animals, both raw and boiled, that the whole white portion of the brain is, of course, divided into very small, round, and somewhat flat fibrils, and so evidently visible in the brains of the fish, that if we look at them through the daylight they will look like an ivory comb, or church organs. He says that the tip or head of these fibrils sinks into the cortex (that is, in the outer gray part of the brain) as to extract the matter from which they must be fed*” (Van Diemerbroeck, 1695).

Three and a half centuries later, there is less mystery regarding such a fibrous composition of the brain. Among the neurons inhabiting the gray matter (GM), there are two groups: interneurons and long-projection neurons. The first group includes neurons that remain more or less confined in the GM to connect the other neurons of the GM together. Long-projection neurons have their cell bodies and their dendritic arborization within the GM, but their axons project their information long distances from the cell body. In addition, long-projection neurons are relatively dispersed in the GM. However, when subsequently emerging from the GM, they arrange themselves in fibers, fan out and then regroup themselves to form bundles of fibers. These axons are myelinated all along their path, which gives the path a whitish color. Consequently, white matter (WM) comprised the parts of the nervous tissue that essentially contain long-range bundles of fibers (axons) sheathed with myelin.

Despite the fact that the brain is made up of billions of neurons, and therefore as many axons with a large number of long-range projections, the spatial organization of such a large number of axons that compose the brain WM is far from being anarchic but is composed of densely packed axons organized into fiber tracts, also named bundles or fascicles. These tracts form a complex but well-organized tridimensional architecture within the hemispheres, the brainstem and the spinal cord.

A detailed knowledge of the anatomy of the WM fascicles is crucial for neurosurgical decision-making and is also of great interest for neuroscience studies in light of the emergence of diffusion-weighted magnetic resonance imaging (DWI) and tractography techniques to reveal the structural connectome of the human brain (Sporns, 2013; Jbabdi et al., 2015). Despite numerous algorithmic developments, diffusion tractography still faces important challenges to properly reconstruct WM tracts for the whole brain (Maier-Hein et al., 2017). Previous studies have demonstrated that even when diffusion tractography is combined with the gold standard anatomical tracer injection technique, tractography parameters (Thomas et al., 2014; Aydogan et al., 2018; Sinke et al., 2018), the superficial fiber system (Reveley et al., 2015) and anatomical constraints (Donahue et al., 2016; Aydogan et al., 2018) strongly bias the tractography results. The most current statement about diffusion tractography is the lack of precise ground-truth anatomical knowledge. Such a lack of trajectories of fiber tracts and their origins as well as terminations in the GM makes it difficult to reconstruct reliable whole brain tractograms, which may encompass the entirety of the tract-based WM organization of the human brain.

Therefore, there is a crucial need to benefit from exact definitions of the trajectories and endings of all WM fascicles. Beyond such definitions, questioning the human WM anatomy with DWI-based tractography has mostly revealed a strong heterogeneity in naming the different bundles, especially the long-range association pathways.

In this review, we propose a comprehensive description of the terminology of the main long-range association pathways of the human brain. Then, we propose a new nomenclature, mainly based on a set of rules of topographical organization of these long-range association pathways.

### A Brief History of the Description of the Fascicular Organization of the WM

**Figure 1:**
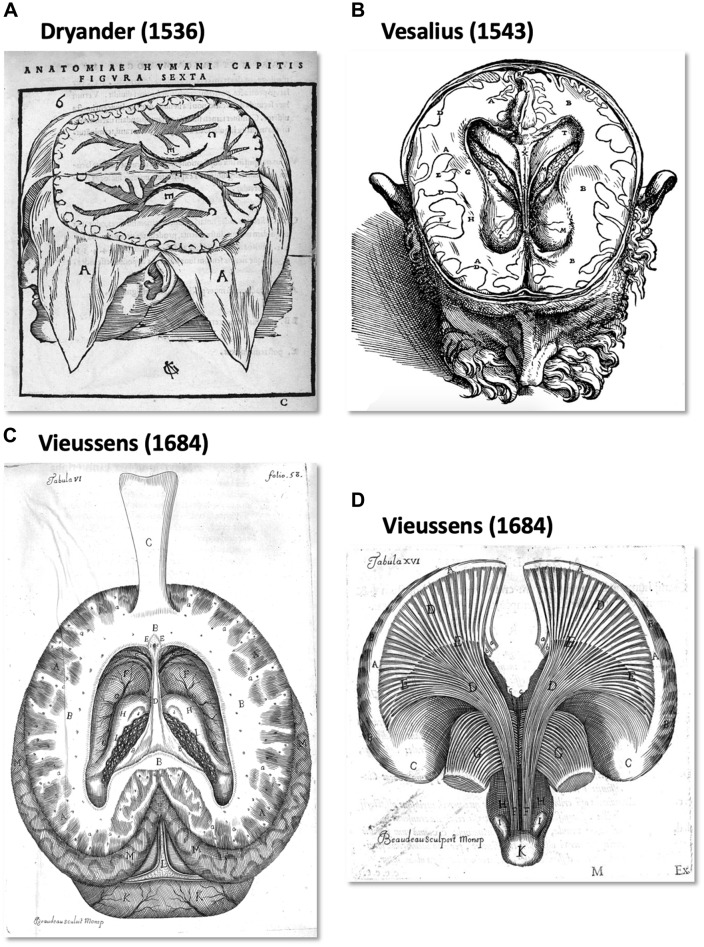
The first illustration of brain anatomy reveals the gross white matter (WM) organization. **(A)** One of the first representations of cerebral circumvolution superficial to the WM in *Anatomica Capitis Humani* (Dryander, 1536). **(B)** Horizontal slice of a human head showing the lateral ventricles, the WM and (less precisely) the gray matter (GM) in *De Humani Corporis Fabrica Libri Septem* (Vesalius, 1543). **(C,D)** Tabula VI and XV of Vieussens’s (1684) *Nevrographia universalis*, respectively. **(C)** A superior section of the human brain at the level of the *centrum ovale* (B) after exposing and folding forward the corpus callosum (C) and **(D)**, the WM tracts projecting from the *centrum ovale* (A) through the *corpora striata* (C,E).

From an historical point of view, the human WM pathways were discretely described by pioneers from the 16th century up to the beginning of the 19th century. In *Anatomia capitis humani* Dryander (1536), (also known as Johann Eichmann (1500–1560)) illustrated the different steps of human head dissection. Figure 1A (Figure 6 from Dryander, 1536) is considered one of the first representations of cerebral circumvolution superficial to the WM (Dryander, 1536). Vesalius provided the same description in *De humani corporis fabrica*, which was published 7 years later (Figure 1B; Vesalius, 1543), and Piccolomini followed by describing a clear distinction between the GM of the cortex and the white medulla (Piccolomini, 1586). Improvements in the specimen preparation during the 17th century allowed finer descriptions (Malpighi, 1665; Steno, 1669; Vieussens, 1684). Steno was one of those who distinguished fiber trajectories within the WM, while as mentioned above, Malpighi also described its fibrous aspect. A significant advance was then made twenty years later when Vieussens found that boiling the brain in oil before dissecting it rendered the WM fibers harder and therefore easier to separate. He discovered that the corpus callosum (Figure 1C) was not a single structure but rather an intricate bundle of fibers that could be separated from the rest of the WM located in each hemisphere.

Due to its unformed appearance upon inspection with the naked eye, the cerebral WM was logically described in terms of large regional patterns (e.g., *centrum semiovale, corona radiata, sagittal stratum, internal, external and extreme capsules*). Vieussens described the WM region located above the lateral ventricles and the corpus callosum, the *centrum semiovale* in relation to its semioval shape (Figure 1D). The *corona radiata*, often referenced as the ventral continuum of the *centrum semiovale*, was considered by Reil as the “longitudinal” component of the WM with a radiating aspect in the sagittal view, in contrast to the “transverse” corpus callosum (Reil, 1809). Mayo translated the Reil description of the corona radiata as follows: “*The fibers derived from the crus cerebri, which diverge at the upper margin of the thalamus towards the circumference of either hemisphere, form the fibrous cone (English term used by Mayo for corona radiata*”; Mayo, 1823). Currently, the *corona radiata* is described as a WM sheet composed of ascending and descending projection fibers, namely, the corticopontine, corticobulbar and corticospinal tracts and the different thalamo-cortical peduncles.

The term “capsule” was also introduced to refer to the bands of WM that pass between cortical and subcortical structures. The internal capsule was first denominated by Burdach (1822) but inspired by Reil (1809), who used the term “*inner wall of capsule*.” It is located between the caudate nucleus and the lenticular nucleus (anterior limb or *crus anterius*) and between the thalamus and the lenticular nucleus (posterior limb or *crus posterius*). The external capsule (EC) is located between the lenticular nucleus and the claustrum, while the extreme capsule is located between the insula and the claustrum (Burdach, 1822).

Beyond this regional terminology, Vicq d’Azir introduced the French term “*faisceau*” (in Latin, “*fasciculus*,” and in English, “*fascicle*” or “*bundle”*) as a cluster of fibers or filaments (Vicq d’Azyr, 1786). Therefore, novel approaches to the gross dissection of brain internal structures led to a new understanding of the WM organization, notably thanks to the works published in the 1800s by Reil (1809), Gall and Spurzheim (1810–1819) and Burdach (1822), highlighting the fascicular organization of the WM. The blunt dissection of fiber bundles performed by Gall and Spurzheim remarkably showed that the WM consists of tracts connecting cortical GM regions that these researchers considered to be the organ of mental activity (Gall and Spurzheim, 1810–1819). It was finally Burdach who defined and designated clearly, through gross dissection studies between 1819 and 1826, the main association pathways, namely, the cingulum, the uncinate fascicle (UF), the arcuate/superior longitudinal fascicle and the inferior longitudinal fascicle (ILF; Burdach, 1819–1826). Subsequently, Meynert made the final distinction between the fibers of association connecting intrahemispheric cortical regions, the fibers of projection connecting a cortical region to a subcortical GM nucleus and the commissural fibers connecting similar regions between both hemispheres (Meynert, 1888).

The enduring anatomical work of Dejerine and Dejerine-Klumpke at the turn of the 20th century describing the WM pathways in detail has therefore been the preeminent authority for over a century (Dejerine and Dejerine-Klumpke, 1895, 1901). Additional association bundles missing in Dejerines’ work were also described early in the 20th century, such as the inferior fronto-occipital fascicle (IFOF; Trolard, 1906; Curran, 1909). The association tract description has therefore been sporadically enriched thanks to the technique of fiber dissection in postmortem human brains, first described by Klingler (Ludwig and Klingler, 1956) and more recently improved (Martino et al., 2011). Based on the freezing of the brains during the fixation process, the cortex-sparing Klingler blunt dissection technique is currently the only technique capable of directly studying the fiber tracts in the human brain at the macroscopic level.

### The Current Status of WM Terminology

Some debates are still ongoing regarding the terminology of the different gyri and sulci of the human cerebral cortex (see in this Research Topic, ten Donkelaar et al., 2018b). However, there is a better consensus for describing the human cortex in terms of gyri and sulci than for describing the WM fiber tracts that link the cortical structures.

The gross dissection, myelin-stained or degeneration techniques used in the 1800s and early 1900s allowed descriptions of the fascicular organization of the WM and the naming of some of the major association bundles either in relation to the cortical structure they connect (e.g., *IFOF, corticospinal tract*) or in relation to their shape (e.g., *UF, arcuate fascicle, cingulum*) and/or their location (e.g., *superior and ILFs*). Table 1 shows how the standard terms used to describe the different bundles have numerous early synonyms and translations that have added to the confusion regarding their description. Although there is still intense debate even about their existence, all these fascicles are named in a confusing way in the current literature, especially with the emergence of DWI-based tractography and the resultantly tremendous increase in WM tract descriptions in the last decade.

**Table 1:** Synonyms and translation of the terms used to describe the main associated fascicles, adapted from Swanson (2015) *Neuroanatomical terminology—A lexicon of Classical Origins and Historical Foundations*.

General name and definition	Earlier synonyms and/or translation
Cingulum (Cing)	- *tenia tecta* (Reil, 1809), Latin form of the term in German, *bedeckten Bänder*, used by Reil; also translated in English as *covered band* (Mayo, 1823); - *lateral longitudinal striae* (Meckel, 1817); - *fillet of the great commissure* (Mayo, 1823); - *peripheral part of the fornix* (Arnold, 1838); - *external fornix* (Arnold, 1838);
External capsule (EC)	- *capsula externa* (Burdach, 1822), original Latin form of the EC first clearly illustrated by Vesalius (1543); - *corporis striati limbus anterior* (Willis, 1672); - *exterior smaller medullary tract of the anterior process of the medulla oblongata* (Vieussens, 1684); - *medullary capsule of the lentiform nucleus* (Arnold, 1838);
Inferior fronto-occipital fascicle (IFOF)	- *inferior longitudinal fascicle* (Trolard, 1906);
Inferior longitudinal fascicle (ILF)	- *fasciculus longitudinalis inferior* (Burdach, 1822), original Latin form of the ILF, perhaps first clearly delineated by Reil (1809); - *longitudinal fascicle arising from the inferior part of the corona radiata* (Arnold, 1838); - *temporo-occipital fasciculus* (Trolard, 1906);
Superior longitudinal fascicle—Arcuate fascicle (SLF/AF)	- *intermediate white matter* (Reil, 1809), first description of a macrodissected adult human SLF; in the original German, *intermediäre Marksubstanz*; - *arcuate fasciculus* (Burdach, 1822); - *longitudinal striae of Reil* (Rolando, 1831); - *lateral longitudinal striae* (Rolando, 1831); - *superior longitudinal commissure* (Solly, 1836); - *longitudinal fascicle of the corona radiata* (Arnold, 1838);
Uncinate fascicle (UF)	- *unciform fascicle* (Reil, 1809), first description of a macrodissected adult human UF; in the original German, *haakenförmige Markbundel*; - *fasciculi unciformes* (Burdach, 1822); - *hamular fasciculus* (Mayo, 1823); - *white nucleus of the Sylvian fossa* (Treviranus and Treviranus, 1816–1821); - *anteromedial arch* (Rolando, 1831); - *olfactory arch* (Rolando, 1831);

Also appearing during the second half of the 19th century but truly emerging as the gold standard for studying brain neuroanatomy at the beginning of the 1970s, the tracing of neural pathways is considered to provide access to the ground truth of structural brain connectivity. Tracer studies inject compounds into the live brain and allow the compounds to disperse by means of axonal transport, marking individual axons over long-range distances. However, these studies can map only a fraction of a neural pathway and are not feasible in humans. A great deal of work has been achieved by such invasive tracing studies in monkeys (Schmahmann and Pandya, 2006). However, a first drift was committed by using the knowledge of the wiring diagram from tract tracing in the monkey as a basis for the classification and functional significance of WM pathways of the human brain (see, for example, Chapter 28 in Schmahmann and Pandya, 2006). A second drift was literally terminological, as some bundles first named in humans based on their shape were also named in the same manner in nonhuman primates but did not show the same shape. In fact, the arcuate fasciculus (AF) was first described and named in humans with regard to the arcuate shape of its fibers connecting the inferior frontal cortex to the caudal superior temporal and middle temporal cortices (Burdach, 1819–1826; Dejerine and Dejerine-Klumpke, 1895). The bundle carrying the fibers from the homologous cortical areas in the macaque monkey has also been denominated the AF but does not show a so-arched shape due to the location and orientation of the temporal lobe in the monkey (Schmahmann and Pandya, 2006; Yeterian et al., 2012). The lack of a consistent connection between the caudal temporal cortex and the inferior frontal cortex in the macaque monkey even led to questioning the existence of the AF in the monkey (Dick and Tremblay, 2012). In fact, as it is now well recognized that, although showing strong similarities, WM pathways in the macaque monkey cannot be considered as the ground truth of human neuroanatomy, especially regarding the association pathways that connect frontal territories, which have undergone a considerably more recent phylogenetic development in humans (Thiebaut de Schotten et al., 2012). As a direct consequence of the odyssey in WM neuroanatomical knowledge and interspecies analogy, many historical denominations of the different bundles are today more confusing than ever. Depending on whether a bundle coexists in monkeys and humans, different names can be used for labeling the same pathways in monkeys and humans, e.g., the extreme capsule vs. the inferior fronto-occipital fasciculus (Schmahmann and Pandya, 2006; Makris and Pandya, 2009). Some previously described association pathways have also been more recently shown to potentially be methodological artifacts. For example, the superior fronto-occipital fasciculus is now considered to not exist in the human brain after having inherited several terminologies from animal studies, namely, the Muratoff or subcallosal fasciculus (Forkel et al., 2014; Meola et al., 2015; Bao et al., 2017). In the same vein, the initial name of a bundle has sometimes been generalized to describe an extension of the initial pathway, without a semantic relationship with the genuine pathway. Indeed, the “vertical portion of the superior longitudinal fasciculus” may be considered as an oxymoron (Bartsch et al., 2013; Martino and García-Porrero, 2013). Finally, new classifications or nomenclatures based on functional rather than anatomical criteria have led to a confusing description of the same anatomical structures. For example, the joint description of the peri-sylvian language pathways vs. the tripartite superior longitudinal fascicles (SLFI, II, III) is more often used to describe WM pathways related to visuo-spatial functions (Catani et al., 2007; Thiebaut de Schotten et al., 2011a,b). The former introduced the description of an anterior segment of the AF (AFas), which carries fronto-parietal fibers that are also considered part of the SLFIII. This led to a confusing situation in which different studies alternatively described their results in terms of AFas or SLFIII depending on whether they dealt with language or spatial functions (Gharabaghi et al., 2009). The AF has also been renamed for some time as the fourth subdivision of the superior longitudinal fasciculi (SLF IV, Makris et al., 2005) but does not have a longitudinal trajectory like those of the three other SLF branches. Hence, progress in our understanding of WM has been hampered by a nomenclature using a wealth of different rules, methods and different species, leading to contradictions and inevitable confusion (see for example the terminology used in the Terminologia Neuroanatomica (TNA; ten Donkelaar et al., 2018a)).

### General Features of the Organization of the Association Pathways of the Human Brain

Association fibers interconnect different cortical areas within the same hemisphere. They are usually subdivided into short and long association fibers. Short association fibers remain within the cortical GM or only pass through the superficial WM between neighboring cortical areas by forming U-shaped fibers around the sulci. Meynert’s pioneering work on WM pathways was the first to differentiate short U-shaped fibers and long association fibers (Meynert, 1892). As specified by the Dejerine” the direction of the U-shaped fibers is always perpendicular to the main axis of the sulcus they cover (Dejerine and Dejerine-Klumpke, 1895). They later reported the following: “*The U-shaped fibers are not generally referred to by a specific name: if, however, for the sake of clarity of the description of a microscopic anatomo-pathological examination, we wish to designate them more especially, it seems to us that the name of the sulcus or the fissure which they cover is the best*.” From a quantitative point of view, the number of U-shaped fibers appears to overwhelm the one of the long-range association fibers in the human brain by at least a factor of 10 (Schuz and Braitenberg, 2002). Although such counts have been estimated based on several assumptions that need confirmation, these authors suggest that only approximately 2% of the total intrahemispheric number of cortico-cortical fibers corresponds to long-rang association fibers, which is the same number as that in the callosal system.

General rules, likely resulting from the biophysics of brain development at the individual level and/or genetic evolution at the species level, have been observed that can help to unravel the complex organization of WM pathways. A debate about the existence of sheet structures in the brain pathways has recently received much attention from the neuroscience and diffusion magnetic resonance imaging communities (Catani et al., 2012a; Wedeen et al., 2012a,b). Wedeen et al. (2012b) proposed that WM fibers form a regular grid by crossing almost orthogonally and uniformly in the entire brain. Although presented as consistent with embryogenesis, such a geometric structure was more mathematically specific than a real characteristic of the brain pathways, and the brain grid theory has not been supported by the evidence (Galinsky and Frank, 2016; Tax et al., 2017). In the same vein, a recent study showed that a dMRI finding thought to be caused by fiber crossings may rather result from sharp turns and/or arborization of fibers than a true crossing between two types of fibers (Mortazavi et al., 2018). Interestingly, and somehow contradicting this claim, Galinsky and Frank (2016) have shown that the overall fiber tract structures of the human brain appear to be more consistent with a small angle treelike branching of tracts forming a lamellar vector field. At the mesoscopic level, this finding is consistent with the laminar origin of cortico-cortical connections demonstrated in nonhuman primates (Barbas, 1986). At the macroscopic level, this propensity for lamellarity in human brain fiber pathways is reminiscent of what the dissectionists “*à la Klingler*” know, and they progress sequentially in a lateromedial direction (Martino et al., 2011). Dissectionists first expose the shorter U-shaped fibers between adjacent gyri, then layer after layer they remove longer fibers between more distant gyri up to the longest bundles (De Benedictis et al., 2012, 2014; Sarubbo et al., 2013, 2016; Fernández-Miranda et al., 2015; Wang et al., 2016; Hau et al., 2017).

Such a practice leads to the consideration of some reliable organizational rules that are useful to apprehend the whole organization of association fibers. One rule is to consider that the long-range fascicles can be defined by their “stem” (Sarubbo et al., 2013; Hau et al., 2016, 2017). The stem is the bottleneck where fibers converge, running all together densely packed over a few centimeters, before fanning in a dispersed manner towards their cortical sites of destination. The stem-based approach applied to diffusion MRI allows virtually dissecting a specific bundle at its densest part, where there are no crossings or too-sharp turns of fibers. In fact, almost all WM regions consist of interdigitated fibers that cross, bend and fan out (Jeurissen et al., 2013; Jones et al., 2013).

Another rule is to consider that the position of the different long association fibers follows a lateromedial organization depending on their length, e.g., the deeper the fibers in the core of the WM, the more distant the two interconnected areas (Curran, 1909; Sarubbo et al., 2016).

## Towards a Common Terminology for Long-Range Association Fibers in Humans

**Figure 2:**
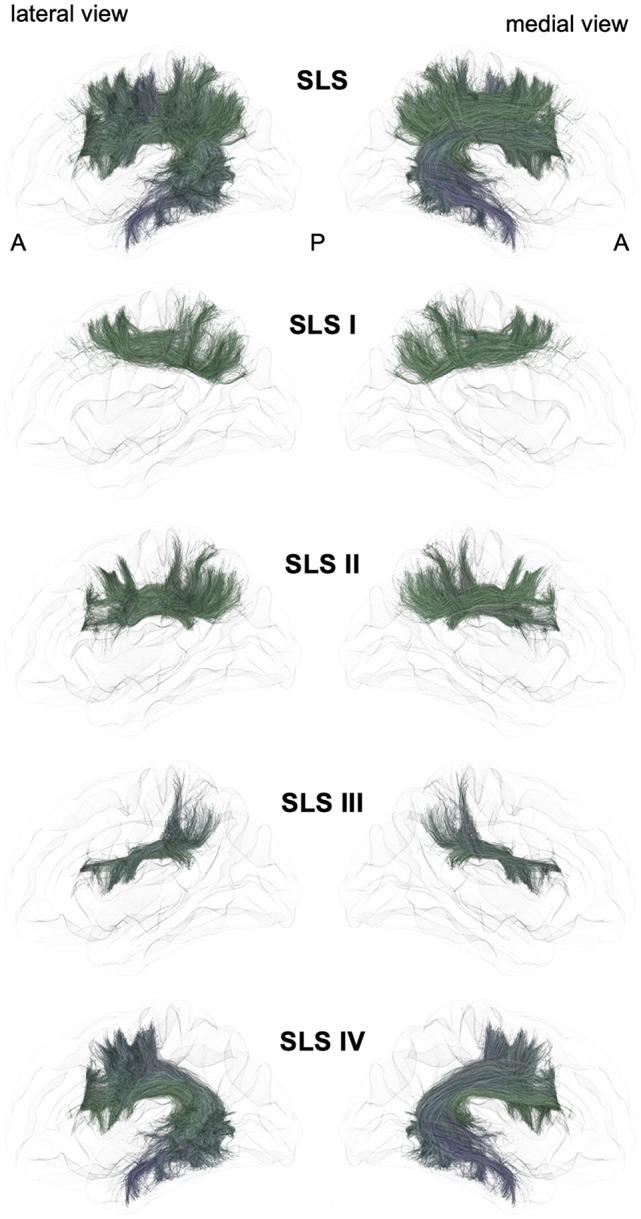
Schematic lateral and medial views of the superior longitudinal system (SLS) and its four different branches. A: anterior, P: posterior. Note that the schematic bundle views were derived from the diffusion tractography data of 42-year-old right-hander male participants of the BIL&GIN database (Mazoyer et al., 2016). Diffusion imaging and whole brain tractography have been detailed in De Benedictis et al. (2016). Briefly, fiber tracking was performed using particle-filter tractography with anatomical priors (Girard et al., 2014) and seeding initiated from the WM/GM interface (10 seeds/voxel). The different association bundles were therefore segmented manually with regions of interest (ROIs) based on the guidelines provided in previous studies (Zhang et al., 2010; Hau et al., 2016, 2017; Rojkova et al., 2016).

**Figure 3:**
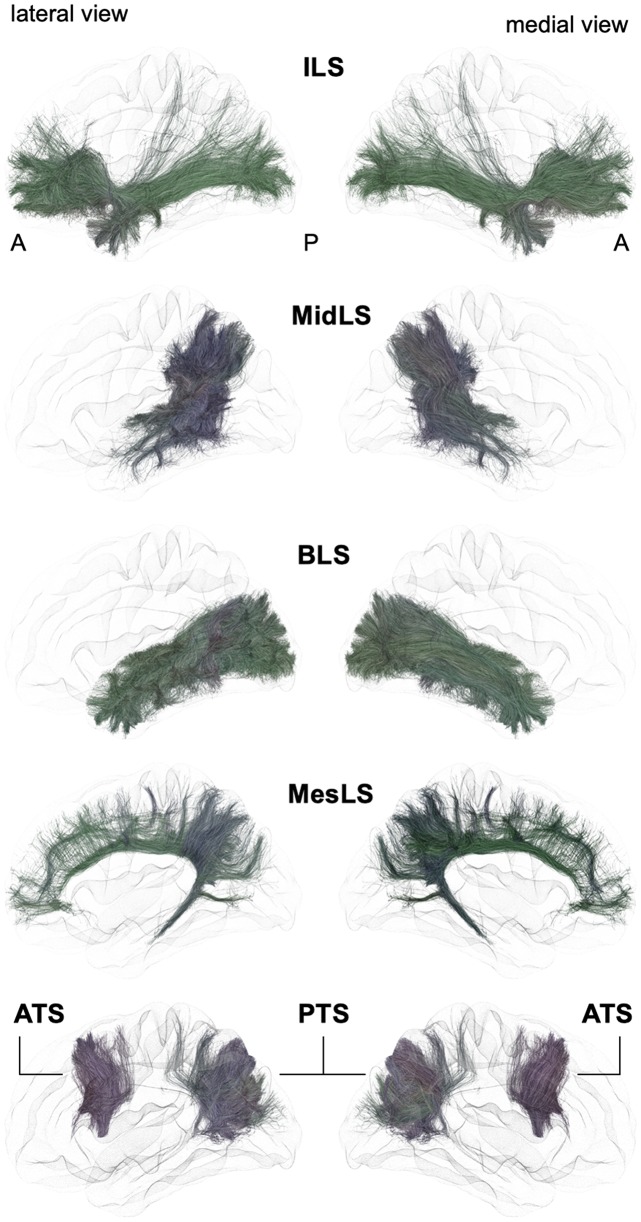
Schematic lateral and medial views of the inferior longitudinal system (ILS), middle longitudinal system (MidLS), basal longitudinal system (BLS), mesial longitudinal system (MesLS), anterior transverse system (ATS) and posterior transverse system (PTS). A: anterior, P: posterior. See Figure 2 for details.

**Figure 4:**
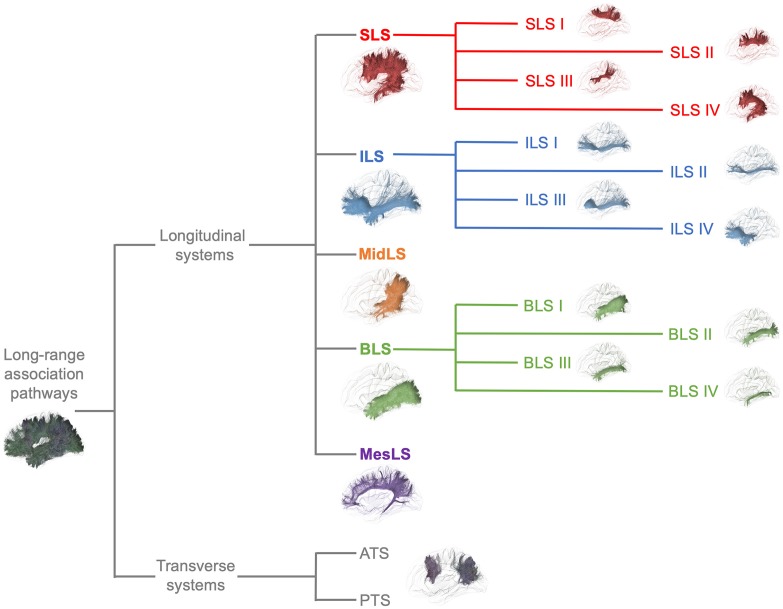
Summary of the proposed nomenclature of the seven main systems of human WM association pathways. The schematic left lateral views of the different systems and sub-systems were derived from the same diffusion tractography data that in Figures 2, 3. See text for details about the terminology and its numbering.

From a general point of view, we propose to classify the long-range association bundles in a hierarchical manner based on the way in which the different distant parts of the same hemisphere can be connected to each other. Due to obvious anatomic constraints, such as the presence of lateral ventricles and subcortical gray nuclei, association fibers connecting one cortical region to another cannot pass anywhere. In fact, both efferent and afferent long-range association fibers connecting the frontal lobe with the parietal, occipital and temporal cortices have only two options of passage, either superiorly at the level of the corona radiata above the superior limiting sulcus of the insula or inferiorly at the level of the inferior limiting sulcus of the insula, within the external/extreme capsule. This situation leads the present stem-based nomenclature to define two major longitudinal systems (superior longitudinal system (SLS), inferior longitudinal system (ILS)) aligned along an antero-posterior axis. Applying the same type of reasoning between each major part of the brain allows us to gather tracts in different systems, according to the global location and orientation of their stems. A second numerical attribute will complement the first level of the hierarchy to more precisely identify the cortical areas connected by the fasciculi. Following this principle, we made an inventory of seven systems that are illustrated in Figures 2, 3 and summarized in Figure 4:

- The superior longitudinal system (SLS)
- The inferior longitudinal system (ILS)
- The middle longitudinal system (MidLS)
- The basal longitudinal system (BLS)
- The mesial longitudinal system (MesLS)
- The anterior transverse system (ATS)
- The posterior transverse system (PTS).

Each of these systems will be now detailed and put in perspective with the current terminology.

### Superior Longitudinal System (SLS, Figure 2)

The SLS gathers the fibers connecting the frontal cortex to the parietal, occipital and temporal cortices by passing through the corona radiata above the superior limiting sulcus of the insula. The SLS comprises the three superior longitudinal fasciculi (SLF I–III) and the AF. Because its stem also belongs to the SLS, the AF is considered to be part of the SLS, albeit the connections of the AF are exclusively fronto-temporal. The fronto-parietal part of the SLS, classically named the “superior longitudinal fasciculus,” has been first described by tracing studies in monkeys (Schmahmann and Pandya, 2006) and described as topographically organized in a very similar way in humans (Thiebaut de Schotten et al., 2011a, 2012; Rojkova et al., 2016; Wang et al., 2016; Parlatini et al., 2017). We will successively detail these fronto-parietal connections of the SLS and then the fronto-temporal connections.

#### The First Branch of the SLS (SLS I)

A dorso-mesial branch of the SLS (Figures 2, 4), joining the superior frontal gyrus with the superior parietal lobe and previously referred to as the first branch of the superior longitudinal fasciculus (SLF I), was first manually delineated on colored-fractional anisotropy maps (Makris et al., 2005). This work was largely inspired by the knowledge coming from monkey tracing studies. However, the described trajectory, running within the gyral WM of the superior frontal gyrus, was shown to be unrealistic (Maldonado et al., 2012). The SLS I was finally defined as the more dorsal and mesial branches of the SLS (Thiebaut de Schotten et al., 2012), connecting the superior frontal gyrus with the superior parietal lobule but running across the corona radiata above the cingulate sulcus. These characteristics allowed differentiating the SLS I from the cingulum, located medially to the corona radiata, below the cingulate sulcus. Hence, the importance of analyzing coronal views when depicting the SLS I cannot be overemphasized: for example, in the study of Kamali et al. (2014), the trajectory of the SLS I corresponds to a branch of the cingulum (Kamali et al., 2014), as also found by Wang et al. (2016). Many other studies are inconclusive regarding the accurate anatomical trajectory of the SLS I, as no coronal slices were provided (Jang and Hong, 2012; Vallar et al., 2014; Yagmurlu et al., 2016; Wang et al., 2016; Fitzgerald et al., 2018). Of note, the stem should also pass below the central sulcus, another property that can be properly analyzed only in the coronal view.

#### The Second Branch of the SLS (SLS II)

The second branch of the SLS (Figures 2, 4) links the middle frontal gyrus with the angular gyrus and posterior part of the supramarginal gyrus and corresponds to the SLF II (Makris and Pandya, 2009; Thiebaut de Schotten et al., 2011a; Wang et al., 2016). Such connectivity is in very good agreement with the findings of electrophysiological connectivity studies relying on the methodology of cortico-cortical evoked-potentials (Matsumoto et al., 2012).

#### The Third Branch of the SLS (SLS III)

The third branch of the SLS (Figures 2, 4) is the most ventral and lateral branch and corresponds to SLF III. It is also the shortest branch, as it links the anterior part of the supramarginal gyrus with the ventral part of the precentral gyrus and the posterior end of the inferior frontal gyrus (Makris and Pandya, 2009; Thiebaut de Schotten et al., 2011a; Wang et al., 2016).

#### The Arcuate Branch of the SLS (SLS IV)

The AF belongs to the SLS because of its stem, which runs parallel and ventrally to the other branches of the SLS (Figures 2, 4). However, the posterior endings of the AF in the temporal lobe dictate its curvature around the posterior insular point, from which its name was derived by pioneering anatomists from Burdach (1822) to Dejerine and Dejerine-Klumpke (1895).

The advent of virtual dissection by diffusion MRI tractography led some authors to gather some short-distance fronto-parietal and parieto-temporal connections under the arcuate terminology. Indeed, a very confusing tractographic study coined the terms “direct” and “indirect” pathways (Catani et al., 2005), both being assigned to the arcuate nomenclature. In fact, the so-called anterior short indirect horizontal segment is none other than the SLF III also defined by the same authors (Thiebaut de Schotten et al., 2011a). It would be anatomically irrelevant to keep the posterior short indirect vertical segment within the arcuate or SLS taxonomy. It should be more naturally referred to as the vertical temporo-parietal fasciculus (VTPF) at the anterior part of the PTS (see below).

A combined fiber dissection and tractography study further subdivided the AF according to the cortical endpoints at both ends (Fernández-Miranda et al., 2015).

#### The Case of the Superior Occipito-Frontal Fasciculus

Finally, to the best of our knowledge, fronto-occipital connections have not been reported within the SLS, except for one study showing SLF connections extending to the most anterior and superior part of the occipital lobe (Forkel et al., 2014). There has been a longstanding controversy regarding the existence of a superior occipito-frontal fasciculus in humans, whose putative stem would have been located, by analogy with the monkey anatomy, at the angle between the corpus callosum and the caudate nucleus, in close relationship with the cortico-striatal tract (also called the subcallosal fasciculus or Muratoff bundle; Schmahmann and Pandya, 2007). The putative SOF has never been evidenced by any dissection studies (Ture et al., 1997; Meola et al., 2015; Bao et al., 2017), but the controversy is still ongoing, and a recent tractographic study suggested that if such a tract existed, it would, rather, be a fronto-parietal one (Bao et al., 2017). In fact, it seems that such a bundle could be a remnant of a fetal pathway that could play a role in axonal guidance during a specific temporal window of brain development, explaining its involution in postnatal brain development and the difficulty in identifying this remnant in adult brains by dissection and tractographic studies (Vasung et al., 2011).

### Inferior Longitudinal System (ILS)

Following the terminology of the SLS, we designate the ILS (Figures 3, 4) as the connections between the frontal cortex and the parietal, occipital and temporal cortices that pass below the level of the inferior limiting sulcus of the insula, within the floor of the external/extreme capsule. This system thus comprises the uncinate fasciculus and the currently named inferior fronto-occipital fasciculus. While the former name was chosen purposely by Reil (1809) to describe the three-dimensional hooked shape of the pathway, the latter inherited a misnomer from its first description. Indeed, in its seminal description, Curran (1909) wrote the following: “*The fasciculus occipito-frontalis inferior is a large associating bundle of fibers uniting, as its name indicates, the occipital with the frontal lobe. It also contains fibers that join the frontal lobe with the posterior part of the temporal and parietal lobes*.” Of note, three years before, the very same fasciculus was coined the “inferior longitudinal fasciculus” by the French anatomist Trolard (1906), who preferred to call the ‘temporo-occipital fasciculus” what is currently termed the “inferior longitudinal fasciculus” (see below the description of the BLS). It should be mentioned that because occipito-frontal connections running through the floor of the external/extreme capsule have never been described in monkeys, the existence of the IFOF has been questioned by authors extrapolating the human anatomy from the monkey anatomy. However, there may be a terminological misunderstanding, and by shedding light on the exact nature of fibers under different names, we may reconcile the two worlds. As mentioned by Curran, the so-misnamed IFOF also includes branches from the frontal lobe towards the caudal part of the temporal cortex. The trajectory of such fibers through the floor of the external/extreme capsule corresponds exactly to the connections named “extreme capsule fiber systems” in monkeys, which are made of fibers joining the frontal and temporal lobes. Moreover, some connections assigned to the middle longitudinal fasciculus in monkeys have been shown to link the frontal and parietal lobes by running through the extreme capsule (Schmahmann and Pandya, 2006), thus making such connections very similar to the fronto-parietal branches of the IFOF. In sum, it seems that the extreme capsule fiber system in monkeys may be conceptually and anatomically part of the IFOF described in humans. However, it must be acknowledged that the existence of true direct occipito-frontal connections in humans remains to be proven by cortico-cortical evoked-potentials, or by any other methodology that would not be subject to false positives, as is the case for gross fiber dissections and tractography in the region lateral to the optic radiations, where it is almost impossible to separate the middle longitudinal fasciculus, inferior longitudinal fasciculus and occipital branches of the presumed IFOF with reliability. From a functional point of view, it is tempting to point out the specific existence of very posterior branches of the IFOF (i.e., in the basal part of the temporo-occipital junction in the fusiform gyrus and in the occipital lobe) in humans as one of the key factors that laid the foundation for the emergence of the human ability to manipulate formal concepts, such as semantic knowledge and its verbal embodiment in language.

In sum, we propose relinquishing the confusing terminology of IFOF and extreme capsule fibers and subsuming under the term “ILS” all fibers coming from the temporal, parietal or occipital lobes, converging at the level of the anterior floor of the external/extreme capsule, and then spreading all over the frontal lobe.

#### The Different Branches of the ILS

For the medio-lateral SLS numerical organization, we propose to distinguish four parts, ILS I–IV, by dividing the frontal terminations of the ILS into four medio-lateral portions (Figure 4).

ILS I–III would encompass the longest fibers connecting ventrally the frontal lobe with the parietal, occipital and temporal cortices, namely, the fibers referred to as the IFOF (Hau et al., 2016; Panesar et al., 2017). The most medial branch with frontal terminations in the most medial third of the frontal lobe would delineate ILS I, while ILS II would comprise the fibers of the inner third of the frontal lobe and ILS III the branches with frontal terminations in the most lateral third of the frontal lobe. There is no current agreement on the posterior terminations of ILS I, II and III (Caverzasi et al., 2014; Hau et al., 2016; Wu et al., 2016; Panesar et al., 2017). Again, this is due to the high degree of overlapping of the posterior terminations of the ILS with the optic radiations, ILF and middle longitudinal fasciculus (MdLF). Moreover, there is also a crossing with the temporo-basal endings of the arcuate fibers.

The fourth branch of the ILS is more commonly referred to as the UF. Most studies agree on its anatomical trajectory, arching around and above the vallecula of the sylvian fissure. Fiber dissection revival started with the work of Ebeling and von Cramon (1992), who detailed different subcomponents, which have also been demonstrated more recently in combined dissection and diffusion tractography studies (Leng et al., 2016; Hau et al., 2017; Panesar et al., 2017).

### Middle Longitudinal System (MidLS, Figure 3)

Contrary to the main association bundles originally macrodissected in humans, the MdLF was first characterized in monkeys. Tracing studies have demonstrated this bidirectional tract, linking the anterior temporal lobe with the inferior parietal lobule. In their monography, Schmahmann and Pandya (2006) also include within the MdLF some fibers linking the lateral and orbital prefrontal cortices with the temporo-parieto-occipital (TPO) area and passing through the extreme capsule. Anatomically speaking, it would have been more coherent to associate such connections with the extreme capsule fiber system, and we assign such connections to the ILS in the new nomenclature (see above).

A diffusion tractography study first reported the existence of the MdLF in humans (Makris et al., 2009, 2013), soon after confirmation by its first dissection (Maldonado et al., 2013) and diffusion tractography studies (Menjot de Champfleur et al., 2013; Wang et al., 2013). The anterior part runs within the WM of the superior temporal gyrus. When reaching the level of the AF posteriorly, the MdLF changes its orientation from the axial plane to the sagittal plane. In its posterior part, the MdLF merges with the deepest fibers of the ILS, rendering both dissection and diffusion tracking complicated. Thus, it is not surprising that there are some discrepancies regarding the posterior cortical terminations of this tract. Most authors agree on the connections with the angular gyrus, while others mainly report connections with the superior parietal lobule and parieto-occipital region (Wang et al., 2013). Interestingly, six different branches of fiber connections of the human MdLF have been recently described, four of which are temporo-parietal and two of which are temporo-occipital (Makris et al., 2017). Following the current nomenclature, they may be considered as different middle longitudinal system (MidLS) branches, but further studies including specific microdissection of these different branches are required before their full description.

### Basal Longitudinal System (BLS, Previously Named “ILF”, Figure 3)

There is a longstanding controversy regarding the so-called “inferior longitudinal fasciculus,” a long association pathway running in the ventral part of the temporal and occipital lobes. Its first description dates back to 1809 by anatomist Reil, but the term “inferior longitudinal fasciculus” was coined by Burdach (1822). Subsequently, in 1906, Trolard coined the term “occipito-temporal pathway.” The existence of this pathway was questioned a few years later (Davis, 1921). Another study combining dissections in humans and radiographic studies in monkeys further argues that the long fibers of the ILF belonging to the external sagittal stratum are nothing but optic radiations (Tusa and Ungerleider, 1985). These authors introduced the concept of the occipito-temporal *system*, which consists of successive series of U-fibers. The first tractographic study focusing on occipito-temporal connections provided evidence for the coexistence of both a series of U-fiber systems and a direct link between the temporal pole to the occipital pole (Catani et al., 2003). At the same time, a fiber dissection study located such direct connections rather inferiorly to the external sagittal stratum, almost within the WM of the fusiform gyrus (Peuskens et al., 2004). Finally, the most recent dissection and tractographic studies subdivided the long-range connections of the occipito-temporal system into several components, according to their posterior occipital terminations (Sarubbo et al., 2016; Latini et al., 2017; Panesar et al., 2018), which follow a multilayered functional organization (Herbet et al., 2018). We thus propose that the present BLS may be subdivided into four branches (BLS I–IV, Figure 4):

– BLS I, linking the temporal cortex to the lateral occipital gyri;
– BLS II, linking the temporal cortex to the cuneus;
– BLS III, linking the temporal cortex to the lingual gyrus;
– BLS IV, linking the temporal cortex within the fusiform gyrus.

### Mesial Longitudinal System (MesLS, Figure 3)

The mesial longitudinal system (MesLS) comprises connections all along the medial surface of the hemisphere, arching from the frontal pole up to the amygdala area. It may essentially comprise two branches:

– Its first branch (MesLS I or “inner cingulum”) corresponds to the cingulum *per se*. There is a wide agreement on the trajectory of this long associative tract, running in the WM of the cingulate gyrus, arching around the splenium of the corpus callosum at the level of the cingulate isthmus, and joining at this level the parahippocampal gyrus, within which it continues its course towards the amygdala.
– The second branch (MesLS II or “outer cingulum”) remains hypothetical. It would correspond to an “outer” part of the anterior cingulum. It has been recently discovered by a diffusion tractography study, which needs additional confirmation from postmortem dissection (David et al., 2018). In essence, such connections are very similar to what some authors call the supracingulate pathway (Wang et al., 2016).

### Anterior Transverse System (ATS, Previously Named “Aslant”, Figure 3)

In 2008, a DTI tractography study described for the first time an associative fiber complex, which interconnects the SMA/pre-SMA of the medial superior frontal cortex to the sus-sylvian precentral gyrus, and the *pars triangularis* and *opercularis* of the inferior frontal gyrus (Lawes et al., 2008). Following this seminal report, this fiber system was further described (Ford et al., 2010; Kinoshita et al., 2012; Vergani et al., 2014a) and finally named the frontal aslant tract (FAT; Catani et al., 2012b; Thiebaut de Schotten et al., 2012). In this latter study, it was also shown that the posterior end of the superior, middle and inferior frontal gyrus constituted a network, with the FAT and U-fibers linking any two pairs of these 3 cortical sites. Following the hierarchical principle of the proposed nomenclature, the ATS can be subdivided into several branches (ATS I–IV), from anterior to posterior, as evidenced by Ford et al. (2010):

– ATS I, first branch of the ATS joining the mesial frontal area to the most anterior part of the pars triangularis;
– ATS II, second branch of the ATS joining the mesial frontal area to the posterior part of the pars triangularis;
– ATS III, third branch of the ATS joining the pre-SMA to the pars opercularis;
– ATS IV, fourth branch of the ATS joining the SMA to the precentral gyrus.

### Posterior Transverse System (PTS, Figure 3)

Mirroring the ATS, the PTS refers to the vertically oriented connectivity, linking the posterior temporo-occipital cortex to the parietal and occipital areas.

The PTS would be composed of two branches (PTS I-II):

– PTS I, located anteriorly, would correspond to connections between the middle temporal gyrus and the supramarginal and angular gyri. PTS I may also be referred to as the *VTPF*, but its inherited name from its first description (Catani et al., 2005), namely, the “posterior short vertical branch of AF,” should not be used anymore. Even if it is indeed located laterally to the temporal part of the arcuate fibers, referring to this tract as a vertical portion of the AF seems rather incoherent. Moreover, recent tractographic studies have revealed some slightly deeper connections, joining the inferior temporal gyrus with the superior parietal lobule (Kamali et al., 2014; Wu et al., 2016). These deeper connections run side by side not only with the vertical part of the deepest arcuate fibers but also with the posterior end of the MdLF.
– PTS II is located more posteriorly and is intralobar. The name *vertical occipital fasciculus* (VOF) could be kept (as it perfectly describes its location and shape), while the historical name of “fasciculus of Wernicke” should no longer be mentioned. This tract has been nicely depicted by fiber dissections (Curran, 1909; Vergani et al., 2014b), as well as by tractographic studies (Yeatman et al., 2014; Wu et al., 2016). It connects the ventral temporo-occipital regions with the transverse occipital sulcus and posterior end of the intraparietal sulcus.

## Conclusion

We propose a new hierarchical nomenclature of long associative intrahemispheric pathways, grouping the tracts into seven main systems designed according to their location and orientation as follows: superior longitudinal, inferior longitudinal, middle longitudinal, basal longitudinal, mesial longitudinal, anterior transverse and posterior transverse (Figure 4). Within each system, the different branches corresponding to distinct cortical endings are listed numerically. However, for some branches, their historical name may be difficult to change (SLS IV, aka AF; ILS IV, aka uncinate fasciculus; MesLS I, aka cingulum; and PTS II aka VOF). We hope that compliance with this new terminology will facilitate the clarity of future studies, especially for newcomers to the field. Finally, the existence of some of the aforementioned branches still needs to be well demonstrated, which will be accomplished by improving the current diffusion tractography tools (Maier-Hein et al., 2017) and combining them with improved cortex-sparing Klingler dissection approaches (De Benedictis et al., 2018), polarized light imaging (Axer et al., 2011), advanced techniques for labeling axon tracts (Brainbow (Weissman and Pan, 2015), and CLARITY (Chang et al., 2017); iDISCO (Renier et al., 2014)) as well as by developing unbiased new methodologies, such as cortico-cortical evoked-potentials (Matsumoto et al., 2012; Mandonnet et al., 2016).

## Author Contributions

EM, SS and LP co-wrote the manuscript.

## Conflict of Interest Statement

The authors declare that the research was conducted in the absence of any commercial or financial relationships that could be construed as a potential conflict of interest.
